# Identification of Key Regulators of Plant Height Variation and Stress Tolerance of the RcMYB Family in *Ricinus communis*

**DOI:** 10.3390/ijms262110318

**Published:** 2025-10-23

**Authors:** Song Yu, Hanhui Wang, Xueying Jin, Jixiang Lin

**Affiliations:** 1 State Key Laboratory of Tree Genetics and Breeding, College of Forestry, Northeast Forestry University, Harbin 150040, China; yusong@nefu.edu.cn (S.Y.); 18846938745@163.com (H.W.);; 2 College of Life Science, Northeast Forestry University, Harbin 150040, China; 3 College of Landscape Architecture, Northeast Forestry University, Harbin 150040, China

**Keywords:** *R. communis*, RcMYB family, plant height, stress tolerance, height-/stress-related RcMYBs

## Abstract

*Ricinus communis* is a significant economic crop, where plant height and stress tolerance are critical factors influencing both yield and quality. The variation in plant height is influenced by both genetic and environmental factors, with environmental stresses such as salt, drought, and cold notably affecting plant growth phenotypes. In this study, we utilized transcriptome data from two varieties, DL01 and Hale, which differ in plant height, to systematically identify the RcMYB transcription factor family and screen 12 key *RcMYBs* associated with height variation. We also analyzed the expression patterns of these genes under various stress conditions, including salt, drought, cold, and heat. Notably, these 12 height/stress-related *RcMYB* genes such as *RcMYB45* and *RcMYB27* showed notable expression changes in response to different stress treatments, suggesting their pivotal roles in regulating both plant height and stress tolerance. Through protein–protein interaction (PPI) network analysis, we further discovered that these RcMYBs could interact with several regulatory factors. This study highlights the roles of RcMYB regulators in controlling plant height and stress adaptation in *R. communis*, providing potential target genes for molecular breeding and offering valuable insights into improving growth performance and stress tolerance.

## 1. Introduction

As a key structural trait, plant height is an important component of crop improvement, directly influencing lodging tolerance and yield performance [1]. Shorter plants, by reducing resource consumption and enhancing lodging tolerance, have been found in short varieties of crops such as *Triticum aestivum* (wheat), *Oryza sativa* (rice), *Zea mays* (maize), and *Sorghum bicolor* (sorghum), as well as in fruit trees like *Malus domestica* (apple) and *Prunus persica* (peach) [2,3,4,5,6]. This is considered one of the results of domestication and selection. However, excessive dwarfing may limit root development and reduce overall stress tolerance. For example, the M9 dwarfing rootstock in apple trees is often less adaptable, with shallow roots that are more susceptible to drought [7]. Plant height influences planting density, and in *Ricinus communis*, while plant density had no significant effect on seed yield in temperate climates, it was positively associated with increased productivity in tropical environments [8]. In addition, the *R. communis* variety Hale shows field tolerance to *Verticillium* wilt and leaf spot, but under salt and drought stress conditions, some tolerant varieties yield 30–60% higher than Hale [9], highlighting the importance of optimizing plant height and enhancing stress tolerance in *R. communis* breeding to improve crop productivity and adaptability. Thus, achieving an optimal plant height and stress tolerance has always been a key objective in breeding.

Disruption of hormone synthesis or signal transduction is the primary cause of plant dwarf phenotypes. Gibberellins (GA) and brassinosteroids (BR) directly affect plant height, with reduced GA levels being associated with dwarfism [10,11,12]. For example, the semi-dwarf phenotype in the IR8 rice variety is caused by a mutation in the *sd1* gene [2], and the dwarfing in the hybrid cultivar DGO24 of *Prunus salicina* L. (plum) is caused by the overexpression of the *PslGA2ox* gene [13]. Additionally, BR plays a crucial role in cell elongation [12], and BR-deficient or insensitive mutants exhibit dwarfism, small leaves, delayed flowering, and other characteristics [14]. GA signaling is a core pathway regulating stem elongation and plant morphology. Studies have shown that specific MYB factors, such as GAMYB, MYB33, and MYB65 in *Arabidopsis thaliana*, mediate GA responses and influence growth-related traits [15,16,17]. Although there are no related reports in *R. communis*, a few genes associated with traits such as plant height, main stem diameter, number of nodes, and seed size have been identified in regions linked to flowering regulation, cell wall synthesis, and adaptability, including the *RcGA2ox2* and *RcMYB46* genes [18]. *AtGA2ox2* controls plant growth and height by regulating GA concentrations [19], while *AtMYB46* is involved in regulating secondary cell wall thickening [20]. However, the functions of these genes in *R. communis* remain unverified. The interaction of these pathways could serve as a promising focus for both experimental research and practical applications.

MYB transcription factors are well-known for their highly conserved MYB domain at the N-terminus, playing a key role in regulating plant height, structural development, lateral organ formation, as well as fruit and seed development [21,22,23]. Previous studies have demonstrated that MYB genes regulate plant height. For example, the *SbMYB110* gene was identified at the *qHT7.1* plant height locus in sorghum, and mutations in *ZmMYB78* in maize significantly affect plant height and inflorescence length [4]. In addition, *OsMYB110* in rice has been found to be induced under low phosphorus stress, and mutations lead to a significant increase in plant height without affecting phosphorus uptake [24]. Other transcriptome studies have also identified MYBs associated with plant height regulation, such as in *Capsicum baccatum* [25] and paper mulberry (*Broussonetia papyrifera*) [26], where several differentially expressed MYB genes were found. Although direct studies on regulating MYB genes to alter plant height in *R. communis* and its related species are limited and not well-developed, their successful application in other crops highlights their immense potential. In *R. communis*, the core gene *RcGA2ox4*, which was upregulated under polyethylene glycol-induced oxidative stress, has been identified through WGCNA [27]. This provides important clues for a deeper understanding of the relationship between hormones, plant height, and stress responses in *R. communis*, opening up new research directions for exploring how these factors interact to regulate plant growth and adaptability.

Plants face biological and abiotic stresses during growth, with abiotic stresses like temperature extremes, drought, salinity, and nutrient deficiencies affecting yield and variety. Many transcription factors and genes are involved in plant responses to stress, improving tolerance by activating specific genes, regulating the abscisic acid (ABA) pathway, and enhancing antioxidant capacity. For example, *MdICE1* from the bHLH family, *MbWRKY50* and *VhWRKY44* from the WRKY family, *FvNAC29* from the NAC family, and *FvMYB44* and *VhMYB2* from the MYB family all enhance transgenic plant tolerance to stress by upregulating antioxidant enzyme activity [28,29,30,31,32,33]. In addition to their crucial role in plant development, MYB proteins are also involved in the plant’s response to environmental stresses, as they not only contribute to the biosynthesis of metabolites like flavonoids and anthocyanins, but also play a key role in coping with abiotic stresses such as drought, salt, and cold [34]. In the rice varieties Nagina 22 and IR64, 92 MYBs showed significantly differential expression in seedlings under drought stress; while in *A. thaliana*, more than 40% of MYBs exhibited differential expression in response to drought stress [35]. In addition, MYB factors could regulate plant salt tolerance through multiple pathways, including redox homeostasis, ion efflux, DNA methylation, and ABA signaling pathways [36,37,38,39]. In cold stress, MYB factors have been found to confer cold tolerance by regulating CBF genes, with AtMYB15 interacting with ICE1 to modulate CBF expression, where overexpression reduces freezing tolerance and loss of function enhances it [40]. However, research on the interaction of MYBs in different signaling pathways, particularly their roles in plant growth and stress response, remains limited. One example is the overexpression of *GmMYB14* in soybean (*Glycine max*), which, by reducing endogenous BR levels, results in a semi-dwarf and compact plant structure and enhanced drought tolerance under field conditions [41]. These results demonstrate the roles of GmMYB14 in regulating plant structure and drought tolerance. MYBs that regulate both plant height and stress response may have the potential to become a promising gene in molecular breeding.

*R. communis*, a member of the Euphorbiaceae family, is an oilseed crop whose breeding goal is primarily to increase yield [42]. It possesses significant advantages, such as drought tolerance, salt and alkali tolerance, and tolerance to heavy metal pollution. Its oil is widely used in the production of high-temperature lubricants, surfactants, and other important industrial products [43,44,45,46]. With the release of the high-quality reference genome in *R. communis* and the establishment of genetic populations, research utilizing forward genetics approaches to uncover the genetic mechanisms controlling targeted breeding traits has been progressively reported [18,47,48]. As a result, most existing research on *R. communis* focuses on analyzing seed yield, oil content, and other components [18,47,49]. For example, the analysis of the hybrid genetic population between the cultivated variety DL01 and the wild type WH11 identified a major locus, miR396b, which regulates seed size in *R. communis* by inhibiting the expression of *RcGRF4* and modulating Indole-3-acetic acid (IAA) content [49]. However, in *R. communis* research, the concept of an “ideal plant type” has been underexplored, with limited in-depth studies and optimization, especially regarding plant height and branching patterns. Since plant height and stress tolerance are critical agronomic traits, their coordinated improvement could significantly enhance both productivity and adaptability, ultimately enabling stable high yields and more efficient cultivation.

Here, we performed stem RNA-seq on two *R. communis* varieties (DL01, tall; Hale, dwarf) and conducted a genome-wide assessment of the RcMYB family, analyzing its subfamilies, physicochemical properties, conserved domains, and promoter elements. Through the DL01-Hale comparison, we identified differential RcMYB expression as a potential contributor to plant height variation and selected 12 key candidates for further investigation. We then evaluated whether these height-related RcMYBs also showed responses under abiotic stresses, including salt, drought, cold, and heat, where the candidates displayed distinct, condition-responsive expression patterns. Therefore, these 12 height/stress-related RcMYBs may play important roles in regulating both plant height and stress tolerance. Further protein–protein interaction (PPI) network analysis revealed interactions between these RcMYBs and several regulatory factors. Taken together, this stepwise progression from the DL01-Hale comparison to the identification of height/stress-related RcMYBs highlights a targeted set of RcMYBs for subsequent functional studies and potential application in *R. communis* improvement. Although this study mainly relies on bioinformatics analysis, further validation of these RcMYBs will be carried out to explore the molecular mechanisms of RcMYBs and lay the foundation for future research in *R. communis* height and stress regulation.

## 2. Results

### 2.1. Genes Associated with Plant Height Differences Between DL01 and Hale in R. communis

**Figure 1:**
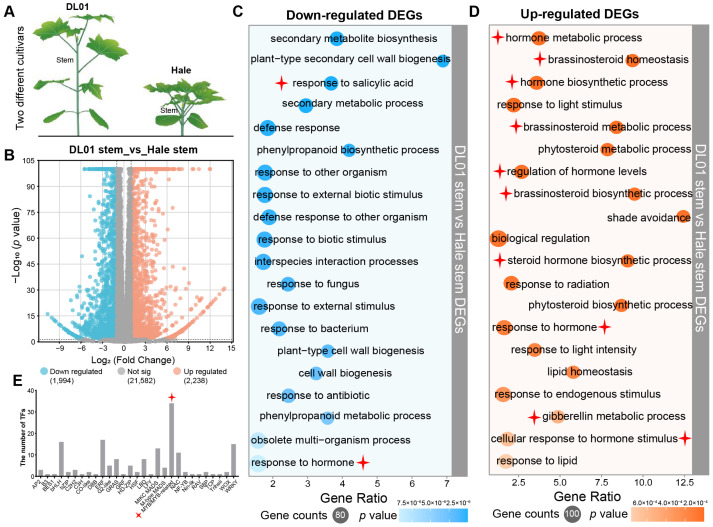
Identification and screening of genes associated with plant height variation in *R*. *communis*. (**A**), Schematic diagram of the DL01 and Hale varieties in *R. communis* showing differences in their stem node regions. (**B**), Volcano plot of differentially expressed genes (DEGs) in the stem node regions of DL01 and Hale. Blue circles indicate downregulated genes in Hale, and orange circles indicate upregulated genes in Hale. (**C**), The top 20 GO enrichment results for downregulated genes. (**D**), The top 20 GO enrichment results for upregulated genes. The color of each circle represents the significance level (*p* value), and the size of the circle reflects the number of enriched genes in each category. (**E**), The number of transcription factor family members in the DEGs, with MYB transcription factors being the most abundant. The star marks indicate the sections that are the main focus of this study.

Two *R. communis* varieties, DL01 (tall plants with long internodes) and Hale (dwarf plants with short internodes), exhibited significant differences in stem morphology (Figure 1A), providing a basis for the identification and functional enrichment analysis of plant height-related genes. Therefore, the stem internodes of these two varieties at the seedling stage were selected for transcriptome sequencing. The results revealed a large number of differentially expressed genes (DEGs) between the two varieties, with 1994 genes downregulated and 2238 genes upregulated in Hale compared to DL01. Gene Ontology (GO) enrichment analysis showed that the downregulated genes were mainly involved in biological processes related to stress responses and cellular structure, including secondary metabolism, response to salicylic acid (SA), defense response, phenylpropanoid biogenesis, cell wall biogenesis, and response to hormone (Figure 1C). In contrast, the upregulated genes were significantly enriched in hormone metabolic processes, particularly in the biosynthesis and homeostasis of brassinosteroids and gibberellins, as well as in light response and lipid homeostasis pathways (Figure 1D).

Notably, transcription factor family analysis indicated that RcMYB family members were the most abundant among the DEGs, suggesting their potential role in the regulation of plant height differences (Figure 1E). Given the obvious differential expression of *RcMYBs* between DL01 and Hale and its central role in plant development and adaptation, *RcMYB* genes were considered key regulators of stem elongation and plant height in *R. communis*.

### 2.2. Identification and Characterization of the RcMYB Gene Family in R. communis

**Figure 2:**
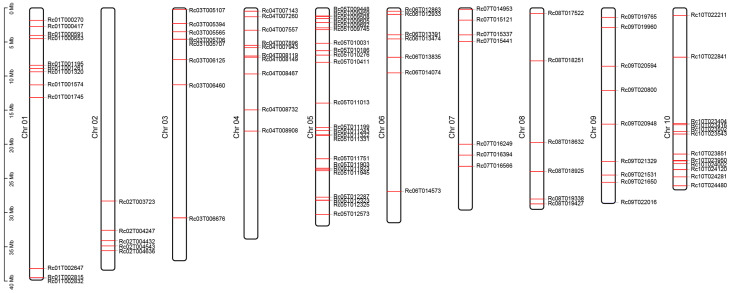
Chromosomal distribution of *RcMYB* members. Chromosome names are labeled on the left side of the chromosomes, and gene positions are marked by red lines.

By downloading 144 MYB family members of *A. thaliana* from the PlantTFDB database [50] and performing BLASTP alignment along with conserved domain analysis, the RcMYB transcription factor family members in *R. communis* were identified (Table S1). A total of 99 *RcMYB* members were identified in the *R. communis* genome, distributed across 10 chromosomes, with the highest number on Chr05 and the lowest on Chr02 (Figure 2 and Table S1). Among them, *RcMYBs* on Chr01, Chr03, Chr05, and Chr06 tend to cluster toward the upper arms of the chromosomes, whereas those on Chr02 and Chr10 tend to cluster toward the lower arms. These results suggested that complex gene duplication of *RcMYBs* and chromosomal rearrangement events may have occurred during evolution. Chromosomal rearrangements such as translocations or inversions may have shaped the present-day organization of *RcMYBs* during the evolutionary history of *R. communis* (Figure 2).

**Figure 3:**
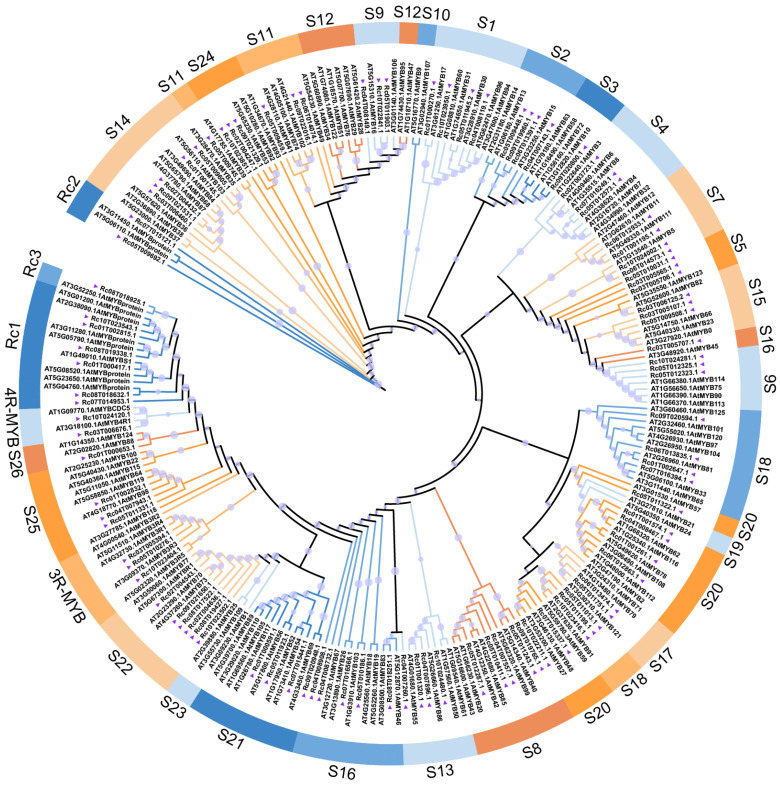
Maximum likelihood (ML) phylogenetic tree of MYB family proteins from *A*. *thaliana* and *R. communis*. The phylogenetic tree was generated with 1000 bootstraps, and bootstrap values are represented by the size of purple circles and displayed next to the nodes. Different colors represent different subfamilies, with subfamily names based on the Arabidopsis subfamily nomenclature. Arabidopsis proteins are represented by ‘AT-’, while *R. communis* proteins by ‘Rc-’ and are indicated by purple triangles.

To explore the evolutionary relationships of RcMYBs, a phylogenetic tree was constructed using the full-length MYB protein sequences from *R. communis* and *A. thaliana* [21] (Figure 3). Based on sequence similarity and clade topology, all MYB proteins were classified into 27 distinct subgroups. The majority of RcMYBs (S1–S26) were assigned to the well-characterized R2R3-MYB class, which is the largest and most functionally diverse subgroup in plants. In addition, smaller but distinct groups corresponding to 3R-MYB and 4R-MYB proteins were also detected, and three novel subgroups (Rc1–Rc3) appeared to be new clades to *R. communis*.

**Figure 4:**
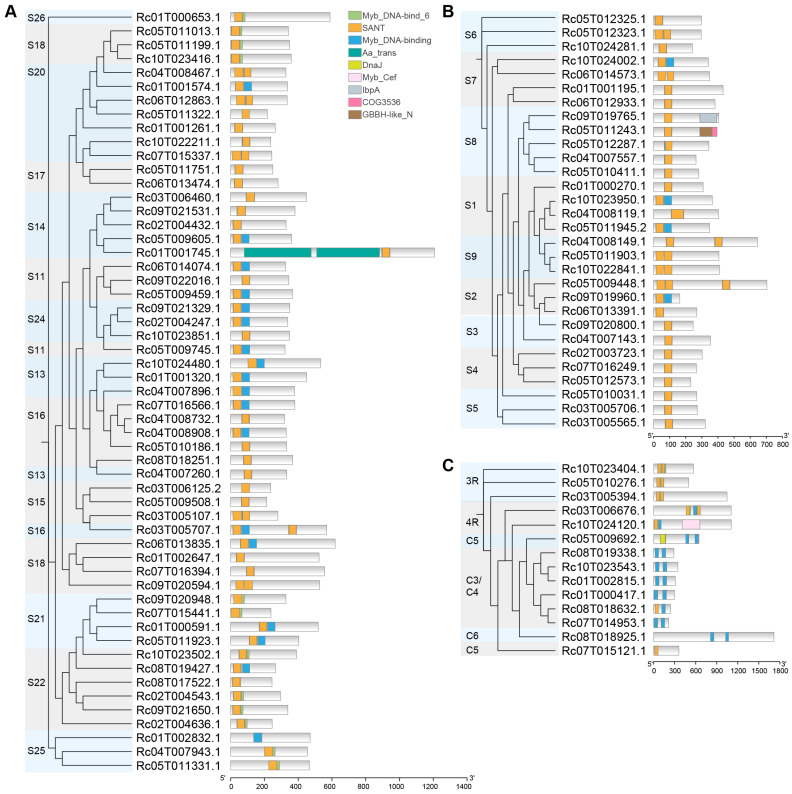
Characteristics of conserved domains in RcMYB family member proteins. (**A**), Phylogenetic tree and conserved domain analysis of the S11–S26 subfamilies. (**B**), Phylogenetic tree and conserved domain analysis of the S1–S9 subfamilies. (**C**), Phylogenetic tree and conserved domain analysis of the 3R, 4R, C3/C4, C5, and C6 subfamilies. Rectangles in different colors represent different domains. Myb_DNA-bind 6, SANT and Myb_DNA-binding domains contain the DNA binding domains from MYB proteins; Aa trans, transmembrane region; IDnaJ domain plays crucial roles in protein translation, folding, unfolding, translocation, and degradation; Myb_Cef is a region of the Myb-Related Cdc5p/Cef1 proteins, and is part of the pre-mRNA splicing factor complex; IbpA belongs to the small heat shock protein IbpA; COG3536, putative dioxygenase with GBBH-like_N/DUF971 domain; GBBH-like N could facilitate dimer formation.

By using the CDD database [51] to predict the conserved domains of RcMYB proteins, a total of nine conserved domains were identified (Figure 4). Most RcMYBs contained the Myb_DNA-bind 6, SANT, and Myb_DNA-binding domains, which showed a certain degree of similarity in their distribution across different proteins. These domains are essential for DNA binding and transcriptional regulation, indicating that most RcMYB proteins may function by directly binding to the promoters of target genes. In contrast, several other domains, including Aa_trans, IDnaJ, Myb_Cef, IbpA, COG3536, and GBBH-like N, were detected only in a subset of RcMYBs (Figure 4A–C), indicating that proteins harboring these motifs may have noncanonical functions.

### 2.3. RcMYB Gene Expression in DL01 and Hale Stems and Plant Height-Related Candidates

**Figure 5:**
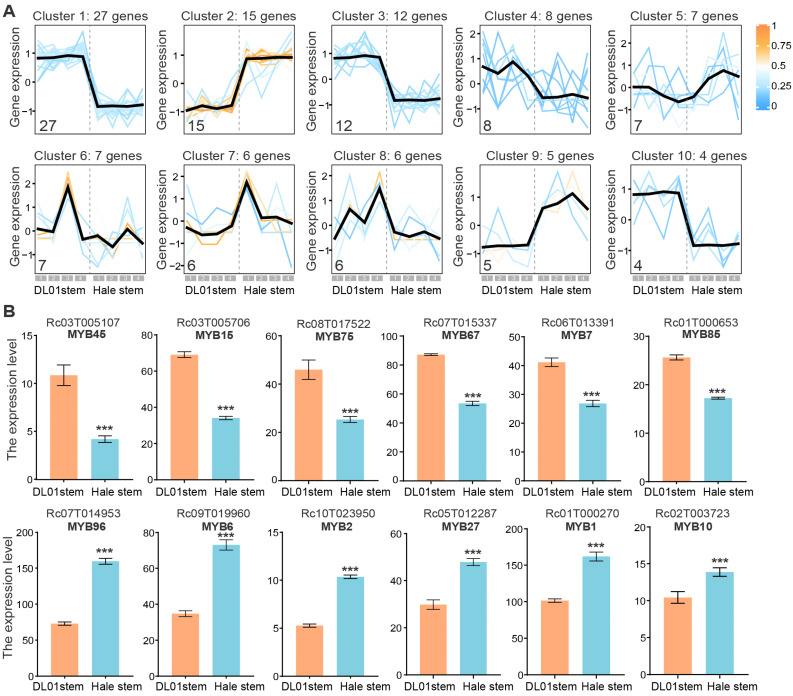
Expression trends and differential expression analysis of *RcMYB* genes in DL01 and Hale stem samples. (**A**), Expression trends of *RcMYB* members in different clusters. The line chart shows the expression trends of different *RcMYB* genes in the stem nodes of DL01 and Hale, with genes clustered based on their expression patterns. The number in the lower left corner of each chart indicates the number of *RcMYB* genes within that cluster. Each line represents the expression trend of each gene, and the color of the line, ranging from blue to yellow, indicates the degree of membership (similarity to the cluster center). The black line represents the average expression trend of all genes within this cluster. The gray boxes at the bottom, labeled 1, 2, 3, and 4, correspond to four sample groups of DL01 and Hale stem samples. (**B**), The top 6 downregulated and upregulated *RcMYB* genes in Hale stem samples compared to DL01, ranked by the fold change in expression. The gene IDs and the names assigned in this study are shown at the top. These genes are referred to as height-related *RcMYB* genes. FPKM values are presented, with error bars representing ± SD of four biological replicates (*n* = 4). Statistical significance was determined by Student’s *t*-test: ***, *p* < 0.001.

To investigate the expression differences in RcMYB genes, we analyzed their expression patterns in the stems of DL01 and Hale. Based on clustering of expression profiles, the *RcMYB* genes were divided into 10 distinct clusters (Figure 5A and Figure S1). Each cluster contained 4–27 genes, with members within the same cluster exhibiting similar expression trends. Several clusters, such as clusters 1, 2, 3, and 10, exhibited notable differences between DL01 and Hale stem samples. Therefore, we focused on the *RcMYBs* that were up- or downregulated between DL01 and Hale, considering them as plant height-related candidates (Figure 5A).

By ranking genes by fold change in descending order and filtering for those with a baseline expression level above 4, we identified the 12 most significantly down- or up-regulated *RcMYB* genes in Hale stems compared to DL01 (Figure 5B). Among them, *RcMYB45*, *RcMYB15*, *RcMYB75*, *RcMYB67*, *RcMYB7*, and *RcMYB85* were significantly downregulated in Hale, while *RcMYB96*, *RcMYB6*, *RcMYB2*, *RcMYB27*, *RcMYB1*, and *RcMYB10* were significantly upregulated (*p* < 0.001). The similar genes in *A. thaliana* are as follows: *RcMYB45*–*AtMYB123*, *RcMYB15*–*AtMYB5*, *RcMYB75*–*AtMYB70*, *RcMYB67*–*AtMYB48*, *RcMYB7*–*AtMYB14*, *RcMYB85*–*AtMYB124*, *RcMYB96*–*AtDIV2*, *RcMYB6*–*AtMYB15*, *RcMYB2*–*AtMYB60*, *RcMYB27*–*AtMYB43*, *RcMYB1*–*AtMYB17*, and *RcMYB10*–*AtMYB3*. The expression changes in these plant height-related *RcMYBs* may play a key role in the regulation of plant height in *R. communis*, particularly in processes such as cell expansion and tolerance responses.

### 2.4. Analysis of Cis-Regulatory Elements and Expression Patterns of Height/Stress-Related RcMYBs Under Stress

**Figure 6:**
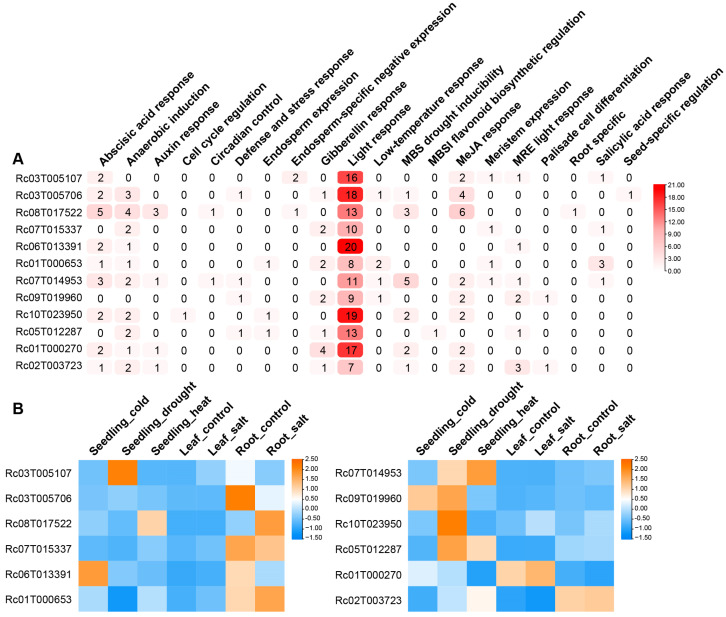
*Cis*-regulatory element analysis of height-related *RcMYB* genes and their expression patterns under stress conditions. (**A**), *Cis*-regulatory elements in the promoter regions of height-related *RcMYB* genes. The numbers represent the count of each element, and the color scale indicates their abundance. The color intensity of each cell, ranging from white to red, reflects the number of elements associated with that gene promoter, as indicated by the color scale on the right. MBS, MYB binding site; MBSI, flavonoid biosynthetic genes regulation, MYB binding site involved in flavonoid biosynthetic genes regulation. (**B**), Heatmaps show the relative expression levels of the top 6 downregulated (left panel) and top 6 upregulated (right panel) *RcMYB* genes under different conditions: seedling under cold treatment (seedling_cold), seedling under drought treatment (seedling_drought), seedling under heat treatment (seedling_heat), leaf with no treatment (leaf_control), leaf under salt treatment (leaf_salt), root with no treatment (root_control), and root under salt treatment (root_salt). Rows represent individual *RcMYB* genes, and columns represent treatments. Relative expression levels in the heatmap are log2-transformed, with blue representing lower expression and orange representing higher expression.

The significant differential expression levels of RcMYB genes between the two *R. communis* varieties highlight their potential role in regulating plant height and suggest they may also help the plant adapt to the environment by modulating stress responses. To further investigate, *cis*-regulatory elements were predicted in the 2000 bp upstream sequences of these genes, and a total of 2290 elements were identified (Figure S2 and Figure 6A). These elements include multiple regulatory factors related to plant growth and stress responses. For example, ABA-responsive elements play a role in drought and salt stress, gibberellin (GA) elements regulate growth and development, while salicylic acid (SA) elements are associated with disease tolerance. The presence of drought and low temperature response elements also indicated the regulatory role of *RcMYBs* under stress conditions (Figure S2). Analysis of the 12 plant-height-related *RcMYB* genes above revealed that their promoters contain different numbers and types of elements (Figure 6A). Elements such as light, hormone, and stress response elements suggested that these genes may be regulated by multiple signals involved in plant growth and stress responses, with their expression potentially varying under different stress conditions.

To further explore the relationship between the regulation of plant height and stress responses by plant height-related *RcMYB* genes, RNA-seq data under different stress conditions in *R. communis* were downloaded. Then we analyzed the expression levels of 6 downregulated and 6 upregulated plant height-related *RcMYB* genes under different stresses, including leaf and root salt stress, heat, drought, and cold stresses during the seedling stage, with untreated leaves and roots as controls (Figure 6B and Figure S3). The results showed that the expression patterns of plant height-related *RcMYB* genes vary considerably under different stress conditions (Figure 6B). Among the downregulated height-related *RcMYB* genes, *RcMYB45* showed increased expression under drought stress during the seedling stage, which may enhance drought tolerance; *RcMYB15* had higher expression in untreated roots, possibly related to normal root development; *RcMYB75* showed increased expression under heat and salt stress in seedlings, likely involved in these adaptive responses; *RcMYB67* exhibited expression changes only under salt stress in roots, potentially regulating root responses to salt accumulation; while *RcMYB7* responded to cold stress in seedlings, possibly playing a role under low temperature stress. Among the upregulated height-related *RcMYB* genes, *RcMYB96* and *RcMYB27* showed increased expression under drought and heat stress in seedlings, while *RcMYB6* exhibited elevated expression under cold and drought stress, indicating the regulatory roles of these genes in response to dual stress. *RcMYB2* responded specifically to drought stress in seedlings, suggesting its potential unique role in drought tolerance regulation. *RcMYB1* was upregulated in leaves under salt stress, while *RcMYB10* showed minor changes in expression under salt stress in roots. Therefore, these plant height-related *RcMYB* genes are closely linked to the mechanisms of plant stress response, making them key functional genes that may play a central bridging role in both plant height regulation and stress adaptation.

### 2.5. PPI Network of Plant Height/Stress-Related RcMYBs

**Figure 7:**
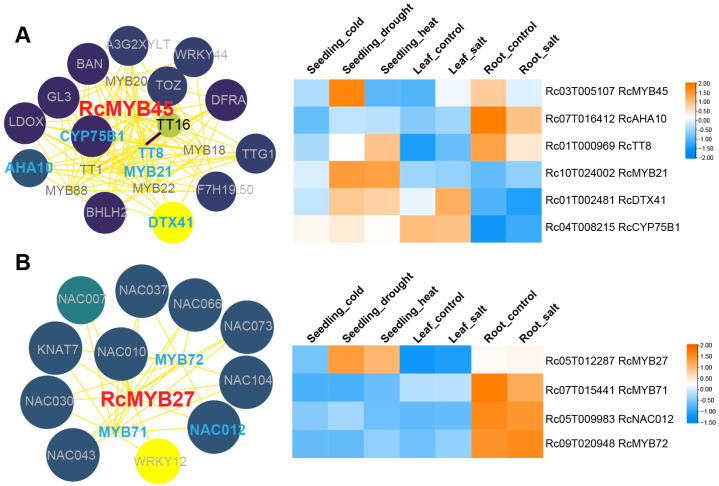
Protein–protein interaction (PPI) network and stress expression analysis of *RcMYB45* and *RcMYB27*. (**A**), PPI network of RcMYB45 and expression analysis of its potential interacting proteins under different stress conditions. The heatmap represents the expression patterns of *RcMYB45* and its height-related interacting genes (in blue text) under different stresses: seedling under cold treatment (seedling_cold), seedling under drought treatment (seedling_drought), seedling under heat treatment (seedling_heat), leaf with no treatment (leaf_control), leaf under salt treatment (leaf_salt), root with no treatment (root_control), and root under salt treatment (root_salt). (**B**), PPI network of RcMYB27 and expression analysis of its potential interacting proteins (in blue text) under different stress conditions. Red text represents the *RcMYB45* and *RcMYB27* genes, while blue text represents their potential interacting genes that show differential expression in DL01 and Hale stem samples. The color intensity and size of the circles represent the confidence of interactions, with darker colors and larger circles indicating higher confidence. The color intensity of the lines represents the strength of interactions, with darker colors indicating a greater potential for interaction.

We selected these 12 genes as height/stress-related *RcMYBs* and performed protein–protein interaction (PPI) screening using the STRING database [52]. The PPI network was optimized by using Cytoscape software v3.8.0 [53], and the retrieved protein IDs were mapped to their corresponding genes (Figure 7 and Figure S4A,B). Interacting proteins with expression differences in plant height between DL01 and Hale samples were highlighted in blue within the PPI network, with a particular focus on the relationship between RcMYBs and these proteins. The greater the number of these blue-highlighted proteins among the interacting proteins, the more prominent the position of these genes in the network. Therefore, in the PPI network, we selected RcMYB45 and RcMYB27, as they interacted with the most blue-highlighted proteins (Figure 7A,B and Figure S4).

RcMYB45 had the highest protein similarity with AtMYB123, which is a key determinant in the accumulation of seed proanthocyanidins [54]. The interacting proteins of RcMYB45 include TT8, RcMYB21, CYP75B1, DTX41/TT12, and AHA10, which are involved in anthocyanin biosynthesis (e.g., TT8, DTX41, AHA10, and CYP75B1), seed coat development (e.g., RcMYB21), and ion transport across the cell membrane (e.g., AHA10) [55,56,57,58] (Figure 7A). When seedlings were subjected to drought stress, the expression levels of RcMYB45, CYP75B1, DTX41, and TT8 were increased; when the roots were exposed to salt stress, the expression levels of RcMYB45, AHA10, and TT8 were increased (Figure 7A). These results indicated that RcMYB45 could regulate plant physiological adaptability and stress tolerance through interactions with different factors.

The most similar protein to RcMYB27 is AtMYB43, which negatively regulates freezing tolerance and participates in the formation of secondary cell walls by directly activating the synthesis genes of lignin and phenylalanine [59,60]. Unlike the PPI network of RcMYB45, factors interacting with RcMYB27 include NAC012/NST3, which is involved in fiber secondary wall biosynthesis; NAC073, KNAT7, RcMYB71 (similar to AtMYB52), and RcMYB72 (similar to AtMYB69), which are associated with secondary cell wall biosynthesis [61,62,63] (Figure 7B). RcMYB27 showed upregulated expression under drought and heat stress conditions during the seedling stages, while its height-related interacting factors exhibited differential expression only under root salt stress (Figure 7B). This difference may be related to the specific regulation of different stress types, stress intensity, or duration. Additionally, the regulatory network of RcMYB27 may involve multiple cross-regulatory factors, so the expression and interaction factor trends could vary under different stress conditions.

## 3. Discussion

Plant height is an important morphological trait, and its regulatory mechanism is related not only to gene expression but also to plant hormones, environmental conditions, and interactions between plants [64]. In *R. communis*, by comparing the stem transcriptomes of the Hale and DL01 varieties, we found that DEGs were predominantly enriched in the BR and GA hormone pathways, suggesting their involvement in the differences in plant height and growth between these varieties (Figure 1C,D). This pattern is similar to the differential expression of some MYBs identified in the long and short varieties of paper mulberry and *C. baccatum*, although the specific members may differ [25,26]. Additionally, the enrichment in pathways associated with secondary metabolism, cell wall synthesis, and hormone response (Figure 1B,C) suggests that MYBs may regulate plant height variation across different species by modulating these pathways.

The MYB gene family is one of the largest transcription factor families in plants, with the R2R3-MYB factors being the most abundant type. In *A. thaliana*, 126 R2R3-MYB genes have been identified, while 244 were found in soybean (*Glycine max*), 117 in rice, 123 in *Salvia nemorosa*, 124 in coconut (*Cocos nucifera* L.), and 65 in quinoa (*Chenopodium quinoa* Willd.) [65,66,67,68,69,70]. In this study, we systematically identified 99 MYB members in the *R. communis* genome, including 85 R2R3-MYBs (Table S1 and Figure 2 and Figure 3). The phylogenetic tree divided the RcMYB members of *R. communis* into 27 subfamilies (Figure 3), all of which clustered with the known functions of similar proteins in *A. thaliana* [21]. The conserved domains in RcMYBs suggested their functional conservation, but variations in the types and number of domains, as well as *cis*-regulatory elements in the promoter regions across subfamilies, may indicate functional differences (Figure 4 and Figure S2).

In this study, 12 height-related *RcMYBs* were first identified (Figure 5); although these genes have not been previously linked to plant height and stress or experimentally validated, they present new targets for further research. *Cis*-regulatory elements and stress treatment analyses also suggested that these genes not only participated in plant height regulation but also could respond to stress signals (Figure 6 and Figure S3). These subtle or tissue-specific expression changes in RcMYBs, while not broadly responsive to stress, may indicate localized regulation in response to specific stress conditions. For instance, RcMYB45 was similar to AtMYB123/AtTT2, which has been reported to be involved in anthocyanin accumulation and heat stress response in *A. thaliana* [54,71]. While *RcMYB45* could contribute to enhanced drought tolerance, its adaptation to heat stress might rely on other regulatory factors (Figure 6). Another gene, RcMYB15, was similar to AtMYB5, which has been shown to enhance heat tolerance when overexpressed [71], whereas RcMYB15 may play a negative regulatory role in the roots under salt stress (Figure 6B and Figure S3). AtMYB70 has been reported to regulate seed germination and root development [72], while RcMYB75 may play a positive role in enhancing root salt tolerance and develop a new function to cope with heat stress (Figure 6). AtDIV, similar to RcMYB96, negatively regulates salt stress by modulating ABA signaling [73]; however, RcMYB96 showed a strong response to seedling drought and heat stress, with only a slight response to salt stress in the roots, possibly due to a higher number of drought-responsive elements in its promoter (Figure 6A,B and Figure S3). Another gene, RcMYB6, responds to cold and drought stress, similar to AtMYB15, whose overexpression can enhance ABA sensitivity and improve drought tolerance [74]. Additionally, AtMYB43 could regulate cold tolerance by modulating the expression of AtCBFs, while RcMYB27 was upregulated under drought and heat stress (Figure 6B), suggesting that despite their similarities, they play distinct roles in adapting to different types of stress. Although these changes may appear subtle or tissue-specific, they could be part of the plant’s adaptive response to complex environmental stresses and hold potential functional significance. These RcMYBs’ functions still need to be validated through further experiments. Future studies should focus on exploring their roles in regulating plant height and stress adaptation, with the goal of uncovering their potential for use in *R. communis* breeding.

PPI network analysis in *R. communis* identified multiple interacting proteins linked to height- and stress-related RcMYBs, suggesting their involvement in the regulation of plant height and stress responses (Figure 7 and Figure S4). RcMYB45 may regulate anthocyanin biosynthesis under drought stress by controlling the expression of CYP75B1, DTX41, and TT8, a mechanism that has been reported in other species, such as wild tomato (*Solanum peruvianum*), *Chaenomeles speciosa*, and common buckwheat (*Fagopyrum esculentum*) [75,76,77]. Additionally, salt stress could cause ion imbalance in plants [78], so RcMYB45 may regulate ion flux to maintain balance and enhance root salt tolerance (Figure 7A). Unlike RcMYB45, the interacting factors of RcMYB27 were primarily associated with secondary cell wall biosynthesis (Figure 7B). AtMYB43 is involved in phenylalanine and lignin biosynthesis, cold stress response, and cadmium tolerance regulation [59,60,79,80], while RcMYB27 was upregulated under drought and heat stress, with significant changes in its interacting factors only under salt stress, suggesting they may help *R. communis* adapt to environmental challenges through specific regulatory networks (Figure 7B).

In addition, the other 10 genes and their interacting proteins exhibited treatment-specific expression patterns: some were induced or repressed under salt stress, some responded markedly to heat or drought stress, while others showed little change under cold conditions (Figure S4A,B,D). The predicted RcMYB-interacting proteins were functionally diverse, also including NAC012, AGL56, LFY, RCMYB52, RCMYB69, RCMYB5, CHX20, AHA10, COR47, RD29A, and XYP7 (Figure S4A,B), playing roles in transcriptional regulation, membrane transport, stress responses, and metabolite trafficking. Although these diverse regulatory networks have not been experimentally validated, they still suggest the functional versatility of these RcMYBs. For instance, the expression patterns of RcMYB6 and its interacting factor RD29A showed that both were upregulated under drought stress, suggesting that they may function in the same drought response mechanism (Figure S4B,D). RD29A, as a typical stress-induced gene, responds more sensitively to drought and cold stress [81], and its interaction with RcMYB6 may directly influence drought tolerance in *R. communis* by modulating key pathways related to stress tolerance (Figure S4A,B). These RcMYBs not only showed differential expression between DL01 and Hale varieties but also exhibited regulation patterns associated with stress conditions (Figure S4). Such tissue-specific and stress-induced expression is common in MYB factors, as observed in related species of *R. communis* such as cassava (*Manihot esculenta*), *Jatropha curcas*, and *Hevea brasiliensis* [82,83,84], where moderate and targeted transcriptional changes are sufficient to activate downstream responses. Although the PPI network was constructed based on predicted results and had limitations due to its reliance on existing databases and algorithms, it could still provide valuable insights for further research on the interactions of RcMYBs and their interaction relationships. The conclusions need to be confirmed through experimental validation.

In summary, through transcriptomic analysis of two *R. communis* varieties with different plant heights (DL01 and Hale), we identified 12 RcMYB factors with different expression levels. These factors were also found to be involved in various stress responses and interact with multiple proteins. These height-/stress-related RcMYBs provide a foundation for further research. We aim to explore the relationship between plant height and stress responses in *R. communis*, particularly by studying these factors, and to uncover their potential mechanisms in regulating plant growth and adaptation to stresses such as salt, cold, drought, and heat. Future work will focus on validating the roles of these RcMYBs through qPCR under different stress conditions, and their functions in plant height and stress responses could be explored using transient overexpression and knockdown experiments. Molecular mechanisms could be further investigated by ChIP-seq for downstream target genes and protein interaction studies using Y2H and BiFC. Due to the lack of a stable transformation system in *R. communis*, transient expression systems may provide an effective alternative for studying regulatory relationships. Despite this limitation, combining transcriptomic analysis and experimental data provides a solid foundation for gene screening and mechanism exploration, aiming to promote molecular breeding and genetic improvement of *R. communis*.

## 4. Materials and Methods

### 4.1. Plant Materials

Two *R. communis* lines, DL01 (tall, long-stemmed) and Hale (dwarf, short-stemmed), were grown in field plots at the Tongliao Academy of Agricultural Sciences (Tongliao, Inner Mongolia, China). Seeds were germinated at 28 °C and, upon emergence, seedlings were promptly transplanted to the field. Conditions were a natural long day (~15 h) with early-summer temperatures of 22–26 °C. Seedling stem internodes from both varieties were collected, immediately frozen in liquid nitrogen, and stored at −80 °C in freezers prior to total RNA extraction. Each sample consisted of 4 biological replicates. The seedling stage was selected to capture active internode elongation, when the DL01-Hale height difference is readily observable.

The stress treatment methods and genotypes were described in [85]. After two weeks of growth in the greenhouse, the seedlings were subjected to one week of drought stress by withholding water. Three-week-old seedlings were used for heat and cold stress treatments. The heat stress group was treated at 45 °C for 12 h, while the cold stress group was treated at 4 °C for 12 h, and whole seedling samples were then collected. For salt stress, two-week-old seedlings were randomly divided into two groups and transferred to a hydroponic system. The control group was continuously supplied with full Hoagland’s solution, while the salt-treated group was supplied with full Hoagland’s solution containing 100 mM NaCl for 24 h. After the treatment, leaves and roots from both the salt-treated and control groups were separately collected.

### 4.2. RNA-Seq and DEGs Identification

Total RNA was extracted using the RNApure Fast Plant Kit (Beijing Cowin Biotech, Beijing, China), and the RNA quality of all samples met the requirements for transcriptome sequencing. RNA samples were subjected to high-throughput sequencing on the Illumina HiSeq 2000 platform. The raw data obtained were quality-controlled using fastp v0.23.4 and FastQC software v0.12.1 [86], then clean reads were aligned using Hisat2 software v2.2.1 and mapped to the *R. communis* reference genome Rc039 [18]. The mapped output was processed by Stringtie to generate counts and FPKM values for all genes in each sample [87]. DEGs were screened using DESeq2, with statistical thresholds set at *p*-value < 0.05 and |log2FoldChange| > 1 [88]. Gene functional annotation of all DEGs was further performed using eggNOG, followed by GO enrichment analysis, and visualization was carried out using TBtools software v2.362 [89]. The classification and quantitative analysis of transcription factors in the DEGs were performed using the PlantTFDB website v5.0 [50].

### 4.3. Identification and Characterization of the RcMYB Family

The MYB family genes and protein sequences of various species were retrieved from the PlantTFDB database [50]. The BLAST tool v2.17.0 was then used for preliminary screening of RcMYB members within the *R. communis* reference genome Rc039. To further refine the candidate genes, HMMER was employed to compare the sequences against the known MYB domain from Pfam [90,91], identifying genes containing the MYB domain in *R. communis*. Domain verification was carried out using the SMART, CDD, and Pfam databases [51,91,92], leading to the identification of the RcMYB gene family. Proteins’ physicochemical properties were analyzed using the Protein Parameter Calculator in TBtools, gene locations on the chromosomes were visualized using the Gene Location Visualizer, the maximum likelihood (ML)-based phylogenetic tree was constructed using the IQtree Wrapper, while domains of each gene were displayed by using the Gene Structure Viewer in TBtools [89].

### 4.4. Analysis of Expression Trends and Cis-Regulatory Elements of RcMYBs

The Mfuzz package in R v4.5.1 [93] was used to analyze the expression trends of *RcMYB* genes and to classify them into clusters, and the expression levels were visualized through a heatmap by using TBtools. Potential *cis*-regulatory elements in the promoter regions of *RcMYBs* were identified using PlantCARE v1.0 [94], and the results were visualized using TBtools [89].

### 4.5. Stress Transcriptome Data Processing and Analysis

The raw stress transcriptome data of *R. communis* were downloaded from the NCBI database [95], including leaf control (SRR21177947, SRR21177959, SRR21177958), leaf salt stress (SRR21177957, SRR21177956, SRR21177955), root control (SRR21177953, SRR21177952, SRR21177951), root salt stress (SRR21177950, SRR21177949, SRR21177948), seedling cold stress (SRR21177961), seedling drought stress (SRR21177960), and seedling heat stress (SRR21177954). Tissue types, treatment conditions, and replicates were assigned based on the metadata provided in the corresponding SRA entries. The raw data were quality-controlled with fastp and FastQC [86], then clean reads were aligned to the *R. communis* reference genome Rc039 using Hisat2. The aligned data were processed by Stringtie to generate gene counts and TPM values for each sample [87]. Gene expression levels were visualized through a heatmap by using TBtools [89].

### 4.6. PPI Network of RcMYBs

The target RcMYB protein sequences were first extracted from the *R. communis* reference genome Rc039, followed by PPI network prediction using the STRING database [52], and potential interacting genes were then identified through BLASTP (https://blast.ncbi.nlm.nih.gov/Blast.cgi (accessed on 10 June 2025)). The network was visualized by using Cytoscape software v3.8.0 [53].

## 5. Conclusions

Through stem-internode transcriptome analysis of two contrasting *R. communis* varieties (DL01, tall; Hale, dwarf), we identified multiple RcMYB transcription factors associated with plant-height regulation. We cataloged the RcMYB gene family, which is widely distributed across the castor genome and exhibits conserved structural features. Those RcMYB genes showing significant differences between DL01 and Hale were prioritized as highly relevant to plant height. Promoter cis-element patterns together with stress-expression analyses indicate regulation by multiple signaling pathways, particularly under abiotic stress. Stress-responsive expression and predicted protein–protein interaction networks suggest that height/stress-related RcMYBs—particularly *RcMYB45* and *RcMYB27*—may interact with multiple regulators. Together, these findings highlight the potential roles of RcMYBs in *R. communis* plant-height regulation and stress adaptation, with the identified candidates showing condition-responsive patterns across salt, drought, cold, and heat datasets and aligning with interaction neighborhoods linked to secondary cell-wall and anthocyanin/ABA-related pathways. These integrated results offer focused targets for subsequent functional studies and for genetic evaluation in breeding materials of *R. communis*.

## Data Availability

All data are contained within the article and the Supplementary Materials, and the generated raw reads have been uploaded to NCBI with GenBank accession numbers of PRJNA1327410. Further inquiries can be directed to the corresponding author.

## Supplementary Materials

The following supporting information can be downloaded at: https://www.mdpi.com/article/10.3390/ijms262110318/s1.
