# Supplementary material for: Identification of Key Regulators of Plant Height Variation and Stress Tolerance of the RcMYB Family in Ricinus communis
Source: Int J Mol Sci. 2025 Oct 23;26(21):10318. doi: 10.3390/ijms262110318 (PMC12607659; doi:10.3390/ijms262110318)
Supplement: Supplementary file 1 [file ijms-26-10318-s001.zip › ijms-3895423-supplementary.pdf]

Table S1 Information of MYB genes in *Ricinus communis*.

PI: isoelectric point; MW: molecular weight.

No.	Gene ID	Gene name	Chromosome localization	Amino acid (aa)	PI	NW	Clade
1	Rc01T000270.1	RcMTB1	Chr1: 1824668-1830182	308	6.99	34134.43	S1
2	Rc10T023950.1	RcMTB2	Chr10: 22375360-22377263	365	6.38	40078.59	S1
3	Rc05T011945.2	RcMTB3	Chr5: 23883845-23886124	348	7.59	38664.08	S1
4	Rc04T008119.1	RcMTB4	Chr4: 7050192-7051977	403	6.15	45278.34	S1
5	Rc05T009448.1	RcMTB5	Chr5: 1185004-1195887	704	4.87	80898.08	S2
6	Rc09T019960.1	RcMTB6	Chr9: 2873544-2876005	163	9.73	18805.53	S2
7	Rc06T013391.1	RcMTB7	Chr6: 3951560-3953496	268	5.22	30491.47	S2
8	Rc04T007143.1	RcMTB8	Chr4: 484752-488132	354	5.18	39905.14	S3
9	Rc09T020800.1	RcMTB9	Chr9: 12106865-12108064	246	6.25	28510.93	S3
10	Rc02T003723.1	RcMTB10	Chr2: 28325484-28327363	302	8.42	34490.54	S4
11	Rc07T016249.1	RcMTB11	Chr7: 20005240-20006710	266	8.94	29912.06	S4
12	Rc05T012573.1	RcMTB12	Chr5: 30292487-30293765	231	9.18	26431.22	S4
13	Rc05T010031.1	RcMTB13	Chr5: 5189617-5190764	268	9.19	30475.55	S5
14	Rc03T005565.1	RcMTB14	Chr3: 3503682-3505132	321	7.98	36311.85	S5
15	Rc03T005706.1	RcMTB15	Chr3: 4586499-4588440	272	7.11	30967.76	S5
16	Rc10T024281.1	RcMTB16	Chr10: 24789958-24791753	241	8.98	27392.23	S6
17	Rc05T012325.1	RcMTB17	Chr5: 28215278-28220490	297	8.44	34213.74	S6
18	Rc05T012323.1	RcMTB18	Chr5: 28185868-28189771	296	5.88	34520.45	S6
19	Rc06T012933.1	RcMTB19	Chr6: 957350-961085	382	5.47	42936.65	S7
20	Rc01T001195.1	RcMTB20	Chr1: 8413104-8417639	432	4.97	48827.67	S7
21	Rc10T024002.1	RcMTB21	Chr10: 22861269-22863524	340	6.9	38566.85	S7
22	Rc06T014573.1	RcMTB22	Chr6: 26929096-26930915	348	8.49	38467.86	S7
23	Rc09T019765.1	RcMTB23	Chr9: 1400478-1403843	405	5.8	47004.11	S8
24	Rc05T011243.1	RcMTB24	Chr5: 18001411-18008488	394	8.95	45084.43	S8
25	Rc04T007557.1	RcMTB25	Chr4: 3274975-3277281	264	6.18	30402.17	S8
26	Rc05T010411.1	RcMTB26	Chr5: 7981615-7983605	281	4.95	32170.67	S8
27	Rc05T012287.1	RcMTB27	Chr5: 27760421-27763062	344	5.01	38817.17	S8

28	Rc10T022841.1	RcMTB28	Chr10: 7228174-7231028	410	6.25	45331.88	S9
29	Rc05T011903.1	RcMTB29	Chr5: 23507938-23510646	406	6.25	44478.02	S9
30	Rc04T008149.1	RcMTB30	Chr4: 7229155-7235765	646	7.05	72840.14	S9
31	Rc05T009745.1	RcMTB31	Chr5: 3196240-3197726	322	6.26	36225.88	S11
32	Rc06T014074.1	RcMTB32	Chr6: 9531611-9533389	325	7.55	36544.1	S11
33	Rc09T022016.1	RcMTB33	Chr9: 28573228-28574771	344	5.56	38840.61	S11
34	Rc05T009459.1	RcMTB34	Chr5: 1256801-1258537	367	5.88	41641.15	S11
35	Rc10T024480.1	RcMTB35	Chr10: 26097135-26099374	533	6.95	59368.27	S13
36	Rc04T007896.1	RcMTB36	Chr4: 5489248-5491132	378	5.89	42984.74	S13
37	Rc01T001320.1	RcMTB37	Chr1: 9376376-9379495	450	6.83	50046.3	S13
38	Rc04T007260.1	RcMTB38	Chr4: 1298963-1301741	333	5.58	37254.56	S13
39	Rc05T009605.1	RcMTB39	Chr5: 2169325-2170919	361	5.99	40183.63	S14
40	Rc01T001745.1	RcMTB40	Chr1: 13120621-13137027	1207	8.31	133655.1	S14
41	Rc02T004432.1	RcMTB41	Chr2: 34100852-34102457	328	8.41	36918.48	S14
42	Rc09T021531.1	RcMTB42	Chr9: 24523768-24526033	381	6.66	42797.08	S14
43	Rc03T006460.1	RcMTB43	Chr3: 11282190-11283963	449	8.03	51028.36	S14
44	Rc03T006125.2	RcMTB44	Chr3: 7599013-7600238	238	8.75	27558.02	S15
45	Rc03T005107.1	RcMTB45	Chr3: 243610-245135	280	4.74	30946.49	S15
46	Rc05T009508.1	RcMTB46	Chr5: 1564172-1566000	214	8.92	24628.69	S15
47	Rc03T005707.1	RcMTB47	Chr3: 4594444-4607212	569	5.98	65086.66	S16
48	Rc08T018251.1	RcMTB48	Chr8: 7769085-7772776	367	5.72	41615.29	S16
49	Rc05T010186.1	RcMTB49	Chr5: 6274194-6276952	332	6.06	37528.81	S16
50	Rc07T016566.1	RcMTB50	Chr7: 23235569-23238897	380	6.2	41891.96	S16
51	Rc04T008732.1	RcMTB51	Chr4: 14961457-14963028	319	6.36	35952.39	S16
52	Rc04T008908.1	RcMTB52	Chr4: 18072430-18073535	329	7.6	37244.85	S16
53	Rc05T011751.1	RcMTB53	Chr5: 22138066-22139022	250	7.83	29319.91	S17
54	Rc06T013474.1	RcMTB54	Chr6: 4558344-4559552	281	9.07	32832.04	S17
55	Rc09T020594.1	RcMTB55	Chr9: 8554264-8557301	527	5.15	57834.55	S18
56	Rc06T013835.1	RcMTB56	Chr6: 7239378-7242857	619	6.73	67953.12	S18
57	Rc01T002647.1	RcMTB57	Chr1: 38164455-38172281	524	5.11	58397.89	S18
58	Rc07T016394.1	RcMTB58	Chr7: 21588780-21594239	556	5.09	60482.31	S18

59	Rc05T011013.1	RcMTB59	Chr5: 13961067-13962095	342	8.91	39257.39	S18
60	Rc05T011199.1	RcMTB60	Chr5: 17553883-17557753	349	9.32	40007.76	S18
61	Rc10T023416.1	RcMTB61	Chr10: 17139425-17143518	359	9.5	41478.57	S18
62	Rc05T011322.1	RcMTB62	Chr5: 18617987-18625670	218	6.53	25021.27	S20
63	Rc01T001574.1	RcMTB63	Chr1: 11292030-11293926	337	6.31	37469.65	S20
64	Rc04T008467.1	RcMTB64	Chr4: 9688864-9690437	327	5.88	37470.96	S20
65	Rc01T001261.1	RcMTB65	Chr1: 8888428-8889977	264	5.12	30389.72	S20
66	Rc06T012863.1	RcMTB66	Chr6: 490866-493164	335	6.31	37486.51	S20
67	Rc07T015337.1	RcMTB67	Chr7: 3958085-3959484	244	8.2	27851.41	S20
68	Rc10T022211.1	RcMTB68	Chr10: 1106208-1107303	238	6.96	27720.34	S20
69	Rc01T000591.1	RcMTB69	Chr1: 4045490-4050170	519	5.8	56737.44	S21
70	Rc05T011923.1	RcMTB70	Chr5: 23657531-23659879	404	8.58	44123.55	S21
71	Rc07T015441.1	RcMTB71	Chr7: 4941599-4944136	239	8.93	28016.21	S21
72	Rc09T020948.1	RcMTB72	Chr9: 17050911-17054872	327	9.28	37057.15	S21
73	Rc02T004543.1	RcMTB73	Chr2: 34881587-34882474	295	8.27	32432.52	S22
74	Rc09T021650.1	RcMTB74	Chr9: 25582954-25583973	339	7.6	36448.65	S22
75	Rc08T017522.1	RcMTB75	Chr8: 826564-827301	245	6.4	27414.63	S22
76	Rc02T004636.1	RcMTB76	Chr2: 35569436-35570176	246	8.49	27030.04	S22
77	Rc08T019427.1	RcMTB77	Chr8: 28745594-28746391	265	5.43	28717.74	S22
78	Rc10T023502.1	RcMTB78	Chr10: 18136233-18139455	390	5.87	42330.32	S22
79	Rc09T021329.1	RcMTB79	Chr9: 22543254-22544719	350	6.42	38828.95	S24
80	Rc02T004247.1	RcMTB80	Chr2: 32584506-32586120	338	5.93	37788.56	S24
81	Rc10T023851.1	RcMTB81	Chr10: 21442840-21444579	349	5.28	39051.01	S24
82	Rc01T002832.1	RcMTB82	Chr1: 39617022-39620094	471	6.1	52361.81	S25
83	Rc04T007943.1	RcMTB83	Chr4: 5822228-5824965	455	6.32	51964.47	S25
84	Rc05T011331.1	RcMTB84	Chr5: 18692585-18695626	467	6	53461.02	S25
85	Rc01T000653.1	RcMTB85	Chr1: 4509545-4516248	589	5.86	65741.82	S26

86	Rc03T005394.1	RcMTB86	Chr3: 2302660-2309495	1046	5.34	115091.5	3R
87	Rc05T010276.1	RcMTB87	Chr5: 6924048-6927648	498	9.24	55939.67	3R
88	Rc10T023404.1	RcMTB88	Chr10: 16978517-16988056	567	8.62	62533.26	3R
89	Rc10T024120.1	RcMTB89	Chr10: 23708614-23721664	1107	5.35	123981.7	4R
90	Rc03T006676.1	RcMTB90	Chr3: 30775393-30788876	1105	8.6	124621.6	4R
91	Rc10T023543.1	RcMTB91	Chr10: 18503461-18506240	344	8.89	38863.75	Rc1
92	Rc01T002815.1	RcMTB92	Chr1: 39512701-39516059	307	9.33	34516.52	Rc1
93	Rc08T019338.1	RcMTB93	Chr8: 28029004-28032558	288	8.92	33240.23	Rc1
94	Rc01T000417.1	RcMTB94	Chr1: 2757066-2758946	297	6.46	32301.07	Rc1
95	Rc08T018632.1	RcMTB95	Chr8: 19735001-19737285	240	6.25	27464.74	Rc1
96	Rc07T014953.1	RcMTB96	Chr7: 208914-209774	214	7.11	24281	Rc1
97	Rc07T015121.1	RcMTB97	Chr7: 1790559-1792880	360	6.66	40540.04	Rc2
98	Rc05T009692.1	RcMTB98	Chr5: 2929298-2932632	647	8.48	74347.85	Rc2
99	Rc08T018925.1	RcMTB99	Chr8: 23982479-23992443	1715	5.81	187739.8	Rc3

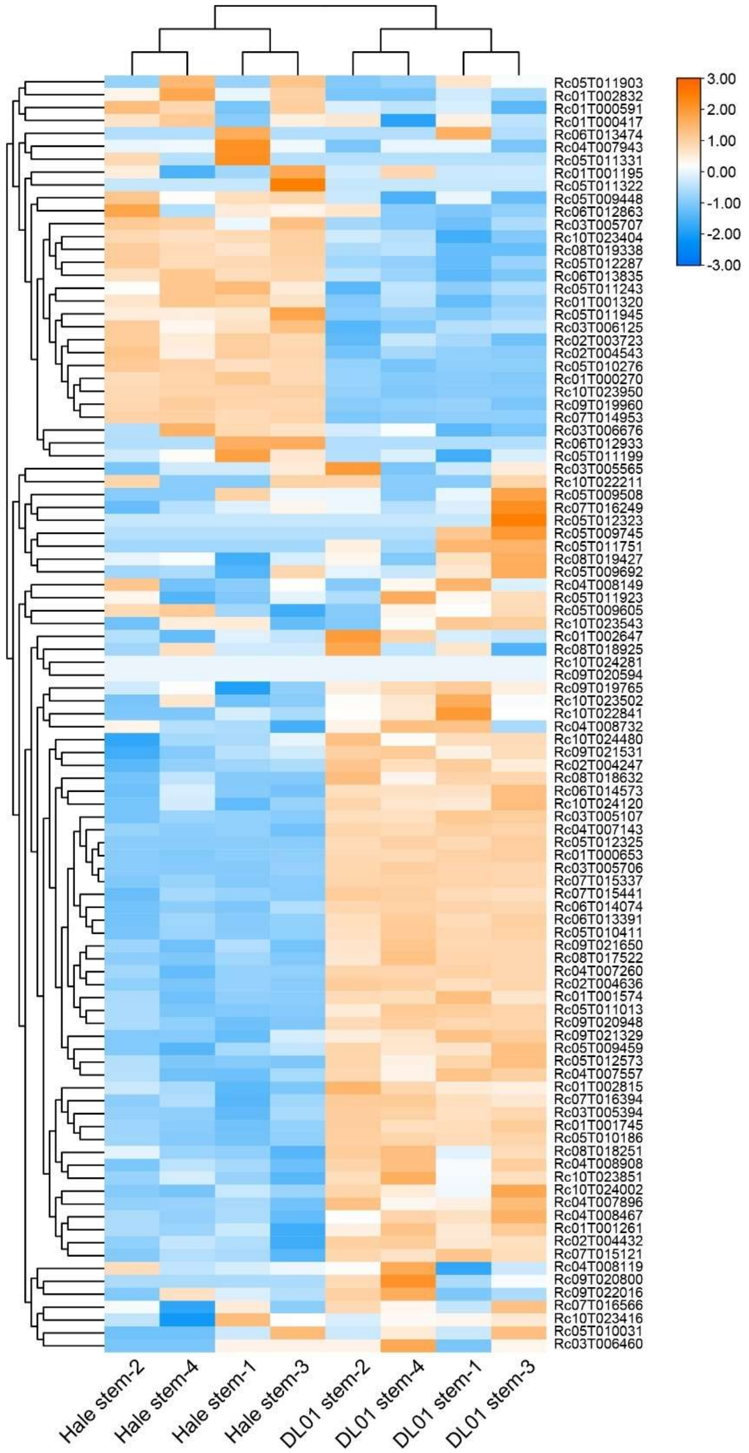

Figure S1 Expression levels of RcMYB family members in the stem nodes of *Ricinus communis* varieties DL01 and Hale.

The relative expression levels in the heatmap were log2-transformed. The color scale represents the relative expression levels, and each sample has 4 biological replicates.

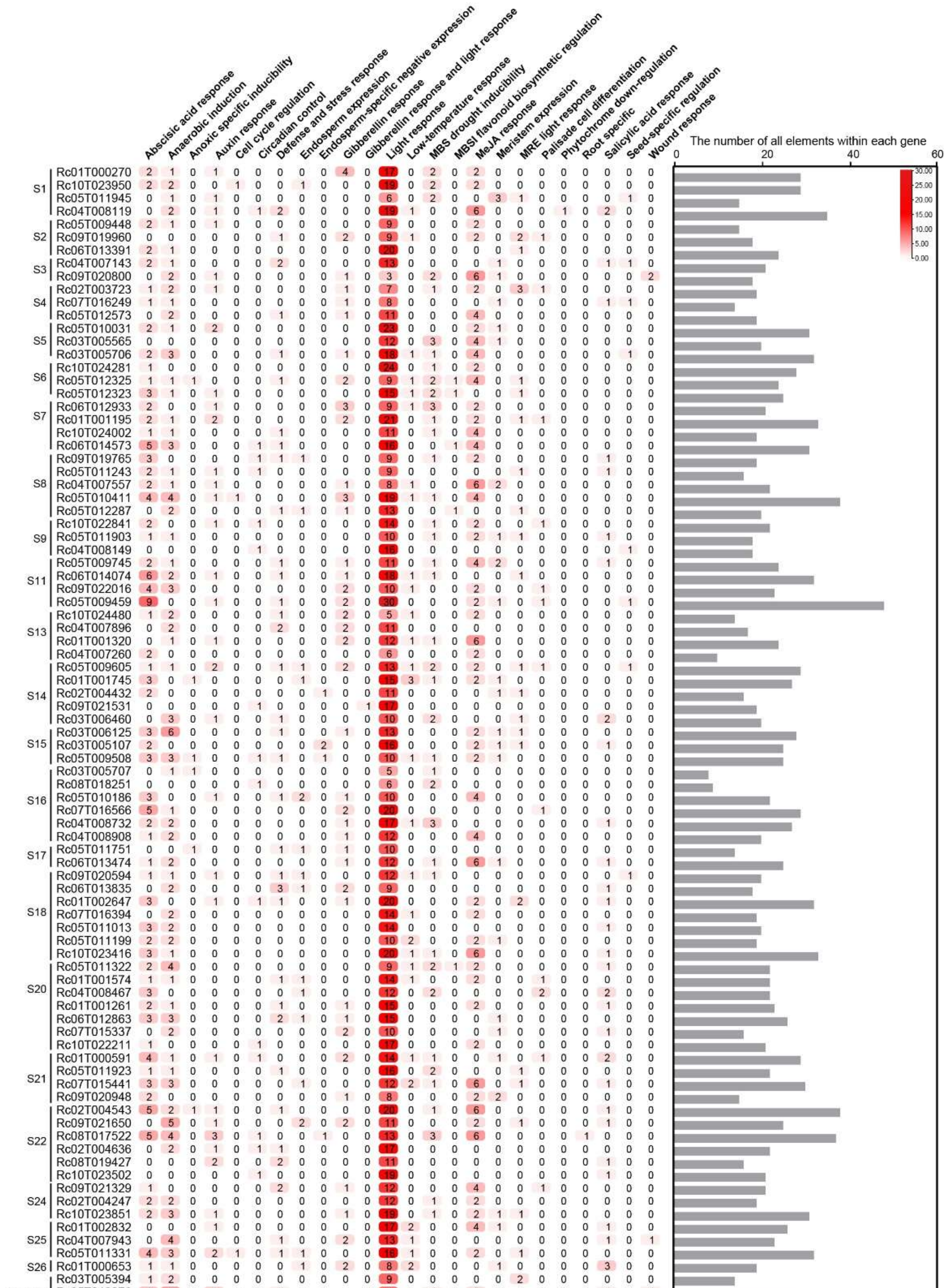

Figure S2 ***Cis*-regulatory element analysis of the promoter regions of *RcMYBs* in *Ricinus communis*.**

The heatmap displays the *cis*-regulatory elements in the promoter regions of *RcMYBs* from different clades. Numbers indicate the quantity of each element, with the color scale reflecting its abundance. The bar chart shows the total number of *cis*-regulatory elements in each gene.

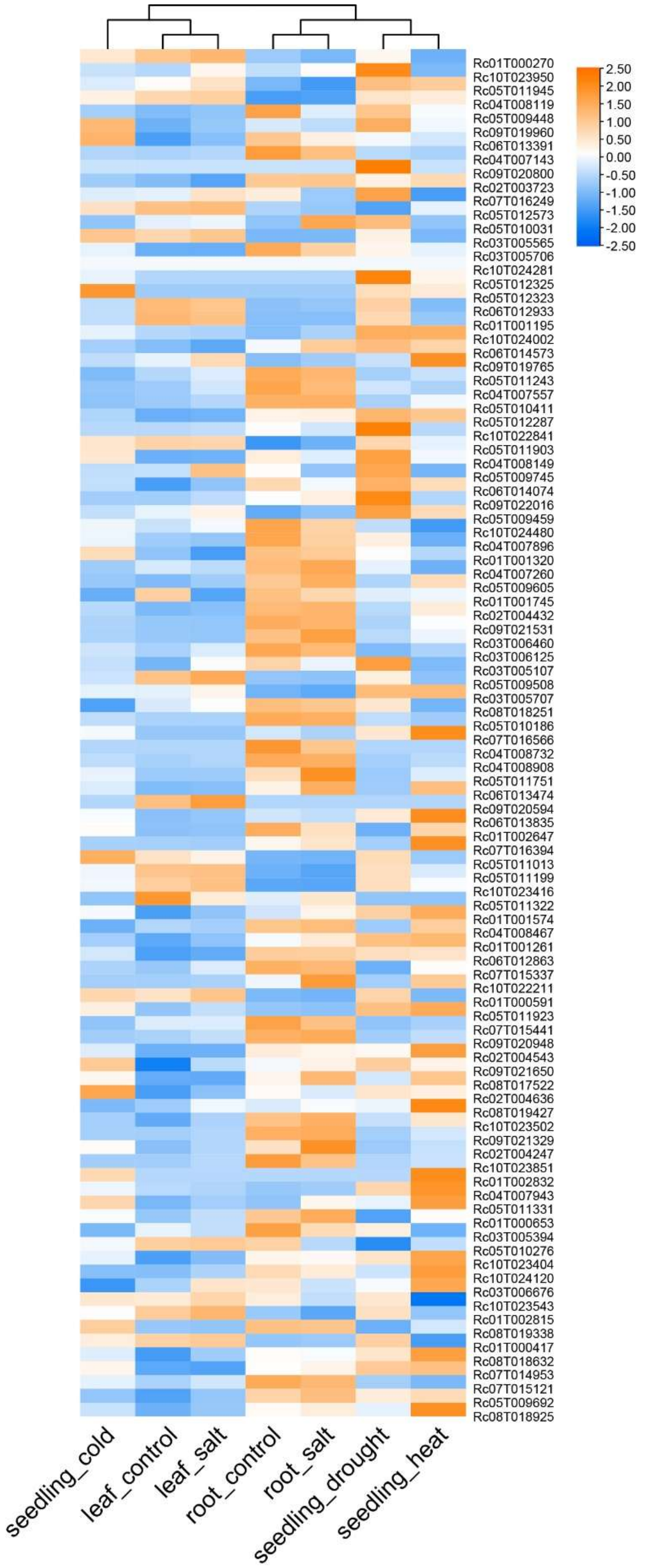

Figure S3 Expression levels of *RcMYBs* under different stress treatments, including salt stress in leaves, salt stress in roots, and heat, drought, and cold stress in seedlings, with untreated leaves and roots as controls.

The relative expression levels in the heatmap were log2-transformed. The color scale represents the relative expression levels.

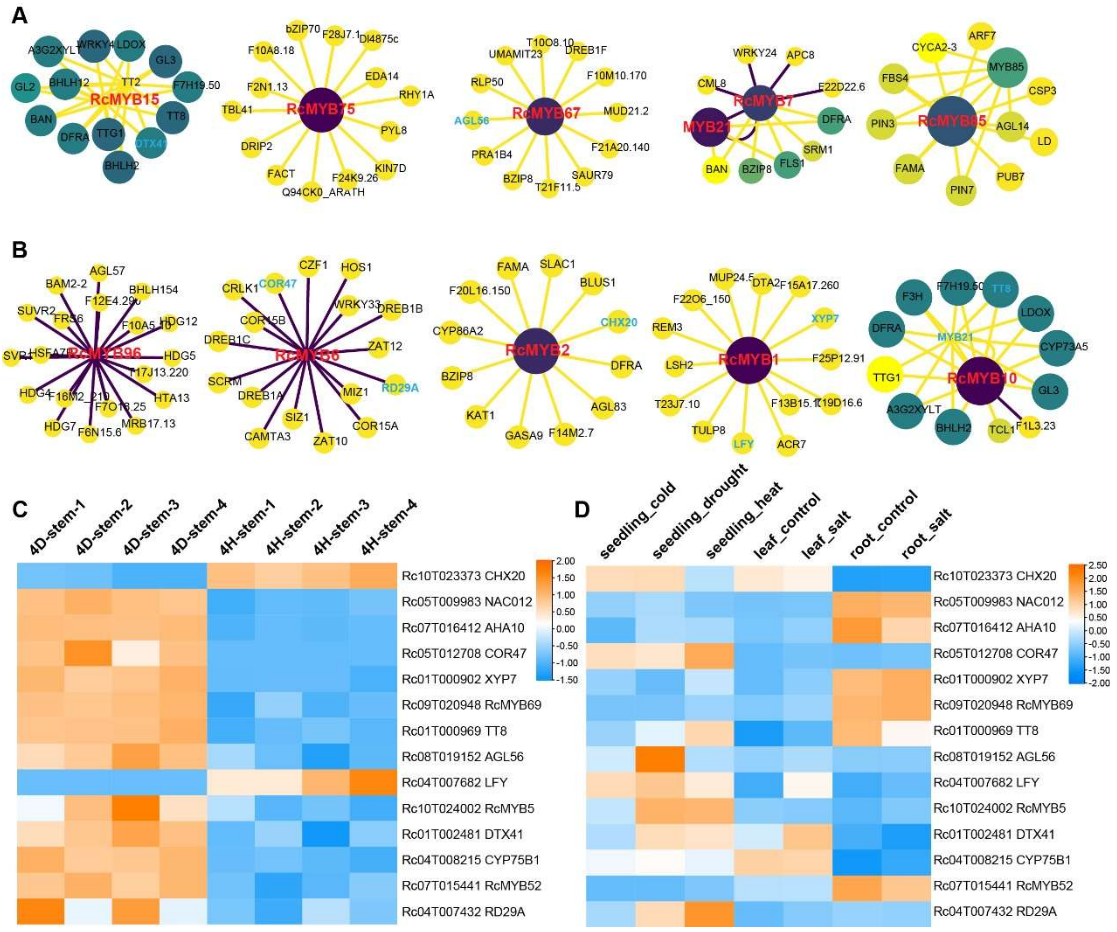

Figure S4 PPI network and stress expression analysis of the height-related *RcMYB* genes.

A, PPI network of the remaining 5 downregulated height-related *RcMYB* genes.

B, PPI network of the remaining 5 upregulated height-related *RcMYB* genes. Red text indicates different *RcMYB* genes, and blue text represents their potential interacting genes that are differentially expressed in DL01 and Hale stem samples. The color intensity and size of the circles represent the confidence score of interactions, with darker colors and larger circles indicating higher confidence. The color intensity of the lines represents the strength of interactions, with darker colors indicating stronger potential interaction abilities.

C, Heatmap of expression levels of potential interacting genes (blue text) in DL01 and Hale stem samples.

D, Heatmap of expression levels of potential interacting genes (blue text) under different stresses.
